# Right Fronto-Subcortical White Matter Microstructure Predicts Cognitive Control Ability on the Go/No-go Task in a Community Sample

**DOI:** 10.3389/fnhum.2018.00127

**Published:** 2018-04-13

**Authors:** Kendra E. Hinton, Benjamin B. Lahey, Victoria Villalta-Gil, Brian D. Boyd, Benjamin C. Yvernault, Katherine B. Werts, Andrew J. Plassard, Brooks Applegate, Neil D. Woodward, Bennett A. Landman, David H. Zald

**Affiliations:** ^1^Department of Psychological Sciences, Vanderbilt University, Nashville, TN, United States; ^2^Department of Public Health Sciences, University of Chicago, Chicago, IL, United States; ^3^Department of Psychiatry and Behavioral Sciences, Vanderbilt University Medical Center, Nashville, TN, United States; ^4^School of Engineering, Vanderbilt University, Nashville, TN, United States; ^5^Department of Educational Leadership, Research and Technology, Western Michigan University, Kalamazoo, MI, United States

**Keywords:** white matter microstructure, general factor, cognitive control, diffusion tensor imaging, response inhibition

## Abstract

Go/no-go tasks are widely used to index cognitive control. This construct has been linked to white matter microstructure in a circuit connecting the right inferior frontal gyrus (IFG), subthalamic nucleus (STN), and pre-supplementary motor area. However, the specificity of this association has not been tested. A general factor of white matter has been identified that is related to processing speed. Given the strong processing speed component in successful performance on the go/no-go task, this general factor could contribute to task performance, but the general factor has often not been accounted for in past studies of cognitive control. Further, studies on cognitive control have generally employed small unrepresentative case-control designs. The present study examined the relationship between go/no-go performance and white matter microstructure in a large community sample of 378 subjects that included participants with a range of both clinical and subclinical nonpsychotic psychopathology. We found that white matter microstructure properties in the right IFG-STN tract significantly predicted task performance, and remained significant after controlling for dimensional psychopathology. The general factor of white matter only reached statistical significance when controlling for dimensional psychopathology. Although the IFG-STN and general factor tracts were highly correlated, when both were included in the model, only the IFG-STN remained a significant predictor of performance. Overall, these findings suggest that while a general factor of white matter can be identified in a young community sample, white matter microstructure properties in the right IFG-STN tract show a specific relationship to cognitive control. The findings highlight the importance of examining both specific and general correlates of cognition, especially in tasks with a speeded component.

## Introduction

The executive function skill of cognitive control, which entails the ability to inhibit inappropriate responses in favor of more appropriate ones, has been consistently implicated in psychopathology (Aichert et al., 2012; Wright et al., 2014). Go/no-go paradigms are frequently used to measure this construct. In a common challenging variation, a series of visual stimuli (X's and Y's) are presented serially, and individuals must make a button press if the current stimulus is different from the preceding one (go trial), and withhold their response if the stimulus is the same as the preceding one (no-go trial) (Garavan et al., 1999). This variant has become popular because of its strong engagement of frontal regions, which reflects the multiple cognitive processes necessary for successful performance (Simmonds et al., 2008).

Neuroimaging studies on cognitive control have often implicated a right lateralized functional neural circuit comprised of the inferior frontal gyrus (IFG), the pre-supplementary motor area (preSMA), and the subthalamic nucleus (STN) (Chambers et al., 2009). This circuit is hypothesized to facilitate the ability to engage in component processes of cognitive control including response selection (selecting between responses) and response inhibition (inhibiting a prepotent response). White matter microstructure plays an important role by determining the quality of communication between regions in this circuit (Aron and Poldrack, 2006). Diffusion tensor imaging (DTI) provides metrics on white matter microstructure, the most commonly reported of which is fractional anisotropy (FA), which broadly looks at how restricted water flow is within white matter, with higher values suggestive of greater restriction, and thus potentially increased efficiency (Basser and Pierpaoli, 1996; Pierpaoli et al., 1996). In particular, higher FA values indicate increased restriction perpendicular to the main axis of the fibers but decreased restriction parallel to the main axis. FA can be decomposed into Radial Diffusivity (RD), which is thought to be more sensitive to properties of myelin, and Axial Diffusivity (AD), which may be more sensitive to properties of axons (Song et al., 2002).

Several DTI studies have found that white matter microstructure properties of tracts connecting the right IFG, preSMA, and STN are associated with cognitive control (Liston et al., 2006; Casey et al., 2007; Madsen et al., 2010; King et al., 2012; Rae et al., 2015). Those studies using the go/no-go task have highlighted the role of both FA and RD in this circuit. However, one notable limitation is that these studies inconsistently test for the anatomical specificity of observed relationships. This is problematic because a substantial amount of the variance in white matter microstructure may reflect more global individual differences that cut across multiple circuits (Penke et al., 2010). Penke and colleagues found that when using a principal components analysis (PCA), FA values across 8 white matter tracts all loaded onto a single factor (2010). Further, that this general factor was associated with processing speed, a construct that is central to the go/no-go task given its emphasis on rapid responses. While this study was in older adults, a general factor has also been identified in younger adults, though the evidence is more mixed (Jahanshad et al., 2013; Johnson et al., 2015; Alloza et al., 2016). Given the potential influence of this general factor in tasks with speeded components, studies should determine if findings are specific to a narrow range of tracts, or related to white matter more broadly. None of the studies to date looking at the relationship between white matter microstructure and go/no-go performance have accounted for this general factor.

It remains unclear the extent to which the relationship between white matter microstructure and cognitive control is specific to tracts connecting the right IFG, STN, and preSMA, vs. linked more globally to the general factor of white matter. Therefore, it is unknown if the previously identified tract-specific relationships could be better explained by this general factor. Such a result would lead to the radically different conclusion that global rather than local white matter microstructure properties facilitate cognitive control. In the present study, we examined the white matter microstructure correlates of go/no-go performance. The primary hypothesis was that cognitive control ability would be selectively related to FA in the circuit connecting the right IFG, STN, and preSMA. The competing hypothesis was that the relationship between cognitive control and FA in this circuit would be explained by the general factor of white matter.

The clinical significance of cognitive control deficits derives in part from their manifestation in psychopathology, especially in externalizing disorders (Young et al., 2009). Studies on white matter microstructure and cognitive control have generally taken a restricted approach to sampling; they often employ convenience samples of super-normals with no history of psychopathology, or compare super-normals to individuals with isolated psychopathology with minimal comorbidity (Kendler, 1990; Cuthbert and Insel, 2013). A significant limitation of these designs is that the findings may not generalize to the general population (Insel et al., 2010). In the present study, we used a large community sample that included individuals with a range of psychopathology. As such, it allowed us to test whether previously identified DTI-performance relationships would generalize to a less restricted sample.

## Materials and methods

### Participants

Participants came from the second wave of the Tennessee Twin Study. The first wave of this study was conducted in 2001 (2000+ twin pairs) and was a representative sample of all live twin births in Tennessee between 1984 and 1995 (Lahey et al., 2008). The now adult twin pairs in the second wave were selected with oversampling for internalizing and externalizing psychopathology risk based on data from clinical interviews when the individuals were adolescents (ages 12–17 in wave one). Thus, the wave 2 sample contains a high proportion of individuals with prevalent forms of psychopathology. Individuals were pre-screened for participation. Exclusion criteria included a history of multiple concussions with loss of consciousness or other head injuries, seizures, neurological diseases other than headaches, contraindications for MRI scanning, diagnosis of schizophrenia, or a major developmental disorder. The study was approved by Vanderbilt University's Institutional Review Board (IRB). Written informed consent was obtained from the participants.

The present sample included 417 young adults (ages 23–31) who completed both a DTI scan and the go/no-go task. A total of 16 subjects were excluded due to poor task performance (defined as performance worse than 2 standard deviations below the average hit rate or two standard deviations above the average false alarm rate) and four subjects were excluded because of a programming error leading to an aberrant ratio of go to no-go trials. We excluded outliers because we could not determine if their poor performance was due to deficits on the task, or to reasons that would invalidate their performance metrics (e.g., using a strategy such as ignoring no-go trials to optimize go performance or they did not understand the directions). In addition, 19 participants were excluded for poor DTI data quality (excessive movement, missing data, etc.).

The final sample consisted of 378 subjects. This included 162 participating twin pairs and 54 individuals without a twin with valid data. For the twin pairs, there were 82 monozygotic pairs and 80 dizygotic (39 same-sex pairs and 41 different-sex pairs).

### Tasks and personality measures

#### Go/No-go task

We utilized the XY go/no-go task developed by Garavan et al. (1999). In this version of the go/no-go task, participants view a series of Xs and Ys, and must respond if a letter is different from the previous one, but withhold their response if the letter is the same. This task was comprised of two 8-min runs, with a total of 732 go trials and 108 no-go trials (total of 840 trials), with go trials being far more frequent than no-go trials in order to make go responses prepotent relative to the less frequent no-go trials. Each trial lasted 1 s, with the letter duration randomly selected (between 600 and 900 ms) and the fixation cross filling the remainder of the time. Prior to completing the task in the scanner, participants were trained on a version of the task that provided feedback on performance. Participants completed the task in a scanner, lying down, and using a response box.

#### Young adult diagnostic interview for children (YA-DISC)

The structured Young Adult Diagnostic Interview for Children (YA-DISC) was administered by a trained interviewer (Shaffer et al., 2000). This computerized structured clinical interview is designed for individuals in the samples' age range and has been used in longitudinal studies of psychopathology to assess symptoms from the major diagnostic categories in the DSM-IV (Hart et al., 1995; Shaffer et al., 1996). The YA-DISC queried diagnostic criteria for attention deficit and hyperactivity disorder (ADHD), oppositional defiant disorder (ODD), conduct disorder (CD), major depressive disorder (MDD), generalized anxiety disorder (GAD), posttraumatic stress disorder (PTSD), agoraphobia, panic attacks, obsessive-compulsive disorder (OCD), social phobia, specific phobia, manic episodes, and nicotine, alcohol, marijuana, and other drug use disorders during the last 12 months. Because far few skip patterns are used in the YA-DISC, it is possible to obtain counts of symptoms for dimensions of psychopathology. For a follow-up analysis, we used a confirmatory analysis to generate both an internalizing and externalizing latent factor score (Lahey et al., 2017).

#### DTI acquisition

Imaging data were acquired on two identical 3T Intera-Achiava Phillips MRI scanners using a 32-channel head coil. T1-weighted images were acquired with a 3-D Magnetization Prepared Rapid Acquisition Gradient Echo (MPRAGE) sequence [TE/TR/TI = 4.6/9.0/644(shortest) ms; SENSE = 2.0; echo train = 131; scan time = 4 min 32 s; FOV: 256 × 256 × 170 mm, 1 mm isotropic resolution]. For diffusion weighted images the scan length was 5 min 2 s. We used a multi-slice Stejskal-Tanner spin echo sequence with an echo planar imaging readout (TE/TR = 52/7750 ms, SENSE = 2.2, FOV: 240 × 240 mm, 2.5 mm isotropic, 50 slices, 2.5 mm slice thickness). This was acquired with one image without diffusion weighting (“b_0_”) and 32 diffusion-weighted images equally distributed over a hemisphere (*b* = 1,000 s/mm^2^).

### Data analysis

#### Behavioral analysis

D-prime served as the primary variable of interest from the go/no-go task. This was calculated by subtracting a z transformation of false alarm rate (the proportion of no-go trials in which the participant incorrectly made a response) from a z transformation of hit rate (The proportion of go trials in which an individual correctly made a response) (Wickens, 2002). In order to complete the z transformation, we used the NORMINV function from Microsoft Excel version 14.6.7. We also calculated inverse efficiency, which is the average go trial reaction time divided by 1 minus the false alarm rate (Townsend and Ashby, 1983). For inverse efficiency, we excluded data for 15 subjects whose scores were 2 standard deviations aberrant from the mean (*n* = 363). SPSS 24 was used to calculate a z transformation.

#### DTI data pre-processing

The DTI data was preprocessed using methods detailed by Lauzon et al. (2013). DTI images were first registered to the B_0_ volume using FSL FLIRT (1). The B_0_ volume was then masked using BET (1). Eddy current and motion corrections were performed using FSL. The CAMINO software package was used to fit the diffusion tensor (Cook et al., 2006). Robust tensor fitting using RESTORE was implemented (Chang et al., 2005). Quality control of the data was completed by consulting a graphical quality assurance report that detailed the amount of motion, FA bias and standard deviation, and goodness of fit of the data to the diffusion model (Lauzon et al., 2013). If a participant was an outlier within the dataset on any of the quality assurance metrics, that participant was excluded from subsequent analyses.

Functional Magnetic Resonance Imaging of the Brain Software Library (FSL;www.fmrib.ox.ac.uk/fsl) was used to compute Tract Based Spatial Statistics (TBSS) and create a white matter skeleton following the procedures detailed in Smith et al. (2006). In brief, each subject's FA image was brought into standard space using a non-linear transformation to the FMRIB58_FA template. These images were then averaged to create a mean FA image, which was then thinned to create a skeletonized mean image and thresholded at *FA* > 0.2. Then each individual's FA image was projected onto this mean skeleton, creating a 4D file that was used for subsequent statistical analyses. Axial diffusivity (AD) and radial diffusivity (RD) skeletonized images were created by first applying the non-linear warp which was used to bring each FA image to the template, and then applying each subject's projection vectors onto the mean skeleton.

### Statistical approach

We used a tract-based approach to look at the relationship between task performance and white matter microstructure across narrow and broad circuits. The first set of tracts of interest were those implicated in cognitive control. For the tracts connecting the right IFG and STN and right preSMA and STN we used masks generously provided by Rae et al. (2015) which were generated via probabilistic tractography in a sample of 16 healthy adults. In addition, we generated a mask of the tract connecting the right IFG and right preSMA. We approximated the methods used by Rae and colleagues by using 16 randomly selected right handed participants (no twin pairs included) who didn't meet criteria for any diagnoses. The IFG mask was from FSL's Juelich histological atlas (BA44 and BA45) and the preSMA/SMA mask was from the anatomical automatic labeling (AAL) atlas (Tzourio-Mazoyer et al., 2002; Eickhoff et al., 2005). We ran probtrackx (FSL) twice with each region as a seed and target, used an exclusion mask at midline, and set target masks as both waypoint and termination masks. Each of these images was thresholded at 98% probability, combined to create a single mask, and transformed to MNI space. Masks for all subjects were then added together and only those voxels that were present in at least 50% of the subjects (8/16) were included in the final mask.

The second set of tracts were those identified as loading heavily onto the general factor of white matter as defined by Penke and colleagues (genu and splenium of the corpus callosum, bilateral cingulum, bilateral uncinate fasciculus, and bilateral arcuate fasciculus) (Penke et al., 2010). We used the JHU white-matter tractography atlas to generate masks for all tracts except for the arcuate fasciculus (AF), which is not included in this atlas (Wakana et al., 2007). For the AF, we used a probabilistic mask generated by the Natbrainlab (http://www.natbrainlab.co.uk/atlas-maps). All masks were thresholded to only include voxels with a probability of >10%.

**Figure 1:**
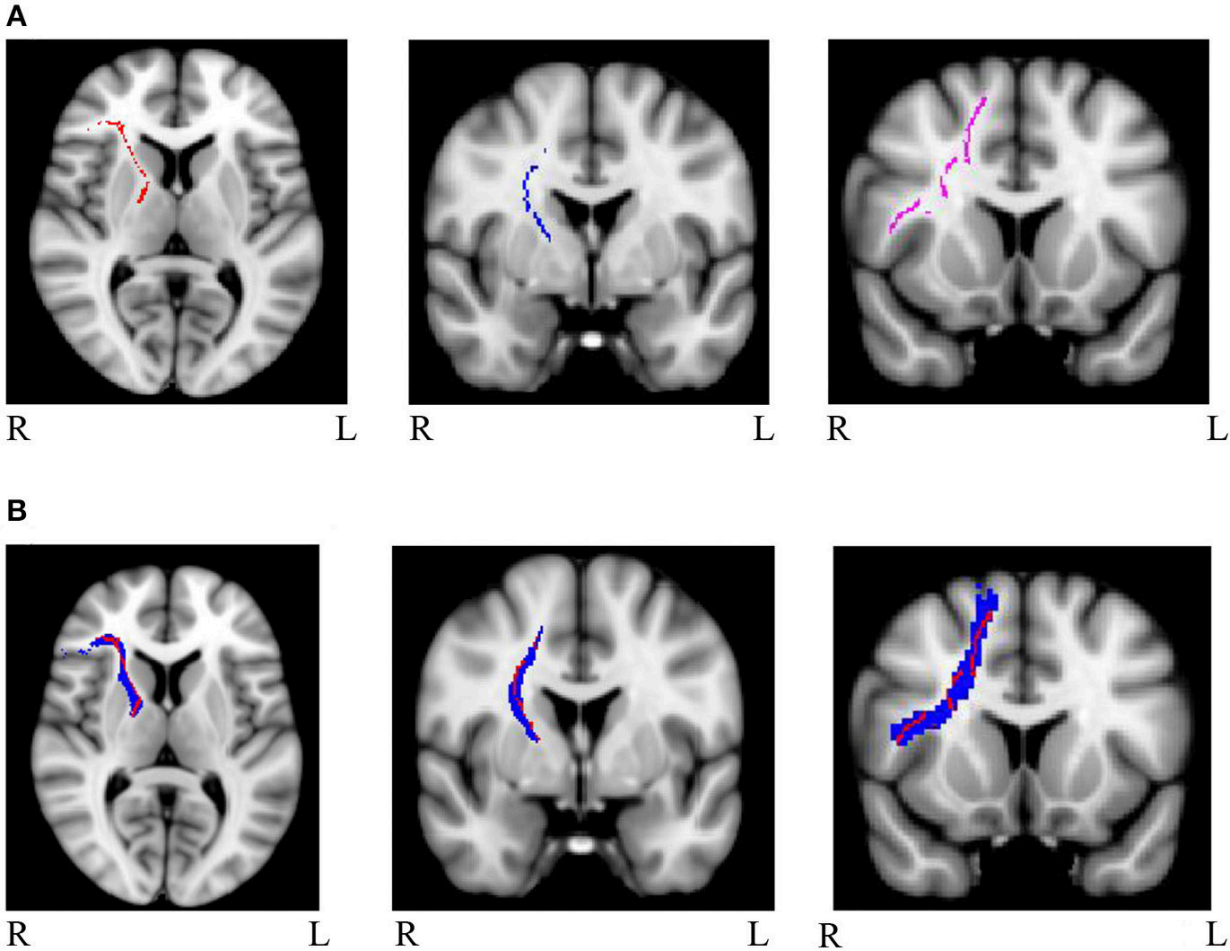
**(A)** Masks of skeletonized response inhibition tracts from left to right: right IFG-STN (red), right preSMA-STN (blue), and right preSMA-IFG (purple) **(B)** Masks of the tracts of interest (in blue) overlapped with the skeletonized images (in red). The tracts are as follows from left to right: right IFG-STN, right preSMA-STN, and right preSMA-IFG.

**Figure 2:**
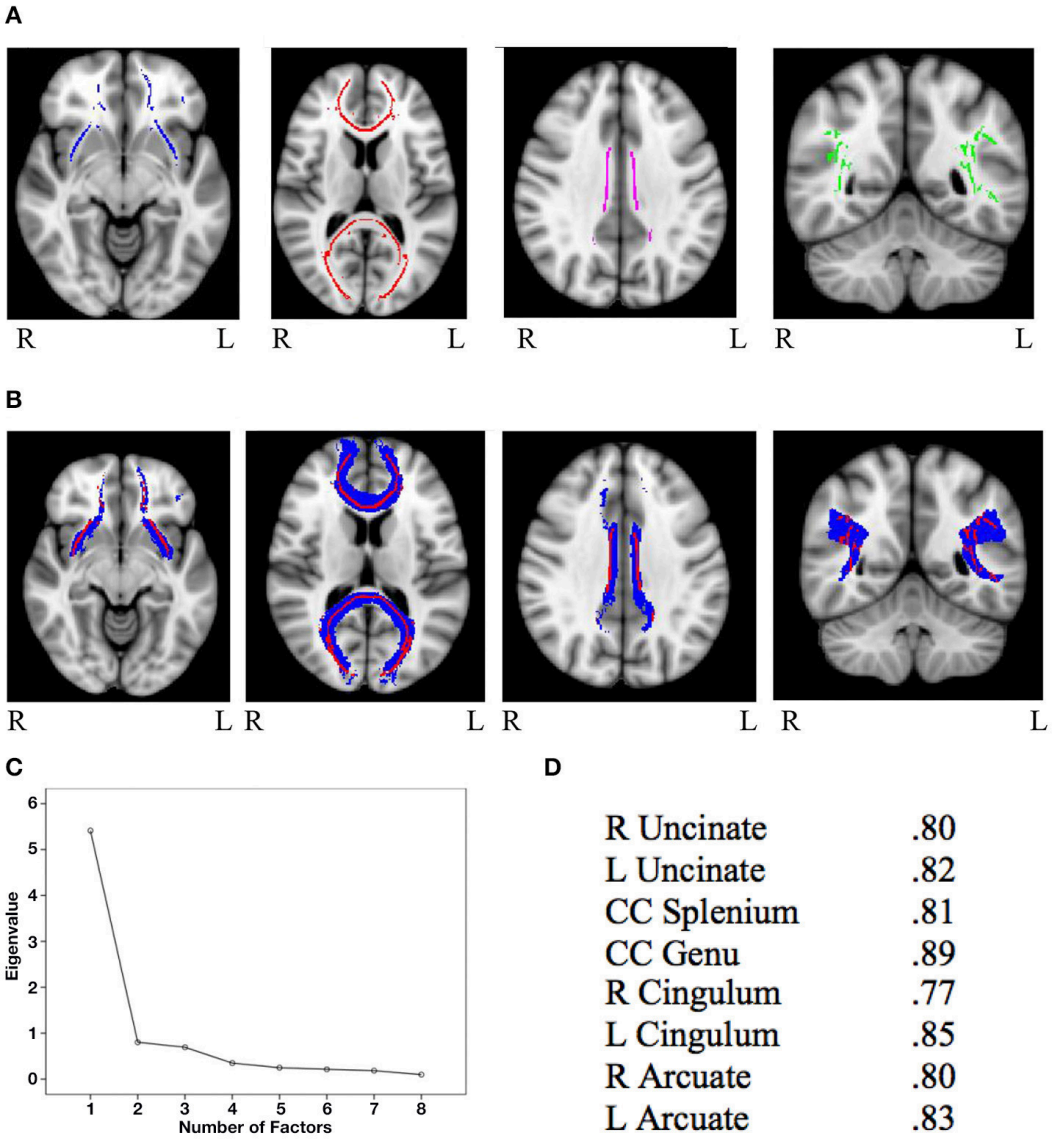
**(A)** General factor skeletonized tracts from left to right: bilateral uncinate fasciculus (blue), splenium and genu of the corpus callosum (red), bilateral cingulum (purple), and bilateral arcuate fasciculus (green) **(B)** Original thresholded tracts (blue) overlaid with skeletonized masks (red). From left to right: uncinate fasciculus, corpus callosum, cingulum, and arcuate fasciculus **(C)** Scree plot from the principal components analysis of FA values from general factor tracts **(D)** Factor loadings of each of the eight general factor tracts.

Mask for the three cognitive control tracts and for the eight general factor tracts were overlaid on the white matter skeleton mask generated from the present sample, and only overlapping voxels were included in the final masks (IFG-STN = 1,289; preSMA-STN = 753; preSMA-IFG = 1,450; genu = 5,491; splenium = 4,092; left cingulum = 934; right cingulum = 511; left uncinate fasciculus = 1,908; right uncinate fasciculus = 907; left arcuate fasciculus = 4078; right arcuate fasciculus = 3,866). See Figure 1A for the skeletonized response inhibition masks and Figure 1B for their overlap with the original tract images. See Figure 2A the skeletonized general factor tracts and Figure 2B with their overlap with the original tract images.

For the response inhibition tract, average FA, RD, and AD values across each mask were extracted for each subject. For the general factor tracts, average FA values were extracted across each of the eight tracts and then a PCA was run on these values using SPSS 24. This method was chosen to be consistent with the methods used in the original general factor of white matter manuscript by Penke et al. (2010). If a single factor solution emerged, factor scores were extracted for each subject and used in subsequent analyses.

To test our hypotheses, we first ran regressions with d-prime as the outcome variable and FA values or factor scores as predictors. Separate regressions were run for general factor, IFG-STN, preSMA-STN, and preSMA-IFG as predictors. We adjusted the *p*-value significance threshold to account for multiple comparisons (0.05/4 = 0.0125). All of the regressions were conducted in MPlus eight and took stratification and clustering within twin pairs into account (Muthén and Muthén, 2012). This included weighting to adjust for both differing probabilities of selection and nonparticipation. Demographic covariates related to the phenotypes included sex, age, ethnicity, and the log of family income during wave one of this study. We also controlled for scanner. To test the power of our analyses we used the program G^*^Power (Faul et al., 2007).

If we observed a significant relationship with both the general factor and one of the specific tracts, we performed a planned analysis to test if relations in tracts of interest would be substantially accounted for by the general factor of white matter. For these analyses, d-prime served as the dependent variable, FA across the significant cognitive control tract(s) as an independent predictor (e.g., IFG-STN/preSMA-STN/preSMA-IFG), and factor scores for FA across general factor tracts as a control variable. For these planned follow-up analyses, we used a significance threshold of *p* < 0.05. Given this is a community sample with a range of psychopathology, we also looked at the role of psychopathology in relation to significant tracts. For these analyses, we used the same statistical thresholds as for the initial analyses.

We also planned a series of analyses to decompose significant relationships. First of all, we looked at inverse efficiency as a measure that examines speed-accuracy trade-offs. We also looked at AD and RD across significant tracts to examine the extent to which properties of myelin or axon may be implicated in cognitive control. We further deconstructed cognitive control performance into response inhibition and selection. For all planned follow-up analyses we used a significance threshold of *p* < 0.05. We also included the same covariates as in the primary analyses (age, sex, ethnicity, log of family income, and scanner) and took stratification and clustering within twin pairs into account and included weighting to adjust for both differing probabilities of selection and nonparticipation.

## Results

### Participant demographics and behavioral results

Table 1 contains demographic information on the full sample, and Table 2 contains information on the number of individuals who met criteria for various diagnoses. A total of 193 subjects did not meet criteria for any diagnoses (51.1%). The average internalizing score was 0.01 (0.22) and externalizing score was 0.09 (0.93).

**Table 1:** Participant demographics and behavioral performance.

**Variable**	**Mean (Standard deviation)**	**Range**
Age (Years)	26.00 (1.75)	23, 31
Income^*^	18.89 (4.94)	1, 24
D-prime	1.48 (0.72)	−0.13, 3.57
Z Hit rate	1.72 (0.56)	0.05, 3.00
Z False alarm rate	0.24 (0.45)	−0.97, 1.38
Inverse efficiency	989.05 (342.69)	460.31, 2014.06
**Variable**	***N***	**Percentage**
Sex		
Male	179	47.35
Female	199	52.65
Scanner		
3TA	199	52.65
3TB	179	47.35
Ethnicity		
White	278	73.54
African american	89	2.35
Other	11	2.91
Psychopathology		
No diagnosis	193	51.06
One or more diagnosis	185	48.94

* *Family income reported in brackets ranging from 0 (no income) to 24 ($100,000 and over). 18 = $35,000-44,999*.

**Table 2:** Participant psychopathology.

**Variable**	***N* (Percentage)**
Alcohol abuse or dependence^*^	76 (20.11)
Antisocial personality disorder	52 (13.76)
Marijuana abuse or dependence	45 (11.90)
Nicotine dependence	33 (8.73)
Specific phobia	30 (7.94)
Social phobia	28 (7.41)
Major depression	24 (6.34)
Agoraphobia	20 (5.30)
Obsessive compulsive disorder	19 (5.03)
Panic disorder	18 (4.76)
Generalized anxiety disorder	17 (4.50)
Attention deficit and hyperactivity disorder	14 (3.70)
Other drug abuse or dependence	11 (2.91)
Posttraumatic stress disorder	10 (2.65)

* *Disorders are not mutually exclusive, and thus individuals may meet criteria for multiple disorders*.

The behavioral metrics from the go/no-go task are also summarized in Table 1. A Komogorov-Smirnov (KS) test for d-prime was not significant, indicating it met assumptions of normality (*p* > 0.10). Hit rate, false alarm rate, and inverse efficiency were not normally distributed (*ps* < 0.05), and thus for subsequent analyses we used the z-transformations of these scores. While z false alarm rate was normally distributed (*p* > 0.10) neither z hit rate nor z inverse efficiency were (*p* < 0.05).

### Principal components analysis

The PCA on the general factor tracts yielded a clear one factor solution, with all 8 tracts loading onto this single factor explaining 67.65% of the variance (for the scree plot see Figure 2C and for factor loadings see Figure 2D). Factor scores were extracted for each subject, and these scores were used in subsequent analyses involving general FA.

### Primary analyses

#### Individual tracts and the general factor

FA in the IFG-STN was a significant predictor of d-prime (β = 0.282, *p* < 0.001). General factor FA was a trend level predictor (β = 0.218, *p* = 0.015), but did not reach statistical significance after correction for multiple comparisons (*p* < 0.0125). FA in the preSMA-STN tract and IFG-STN tract were not significant predictors (*p* > 0.10). The significant regression models are presented in Table 3.

**Table 3:** Significant regression models predicting d-prime.

	**FA IFG-STN**		**FA IFG-STN controlling for psychopathology**		**FA general controlling for psychopathology**	
	**B (SE)**	**β**	**B (SE)**	**β**	**B (SE)**	**β**
FA IFG-STN	8.56 (2.11)^**^	0.28^**^	8.20 (2.07)^**^	0.27^**^	–	–
FA General^a^	–	–	–	–	0.16 (0.07)^*^	0.22^*^
Internalizing	–	–	−0.16 (0.27)	−0.04	−0.10 (0.31)	−0.03
Externalizing	–	–	−0.13 (0.09)	−0.15	−0.16 (0.10)	−0.18
Sex	0.03 (0.12)	0.02	−0.02 (0.12)	−0.02	−0.05 (0.11)	−0.03
Age	0.04 (0.03)	0.08	0.04 (0.03)	0.09	0.04 (0.03)	0.10
Ethnicity	0.21 (0.13)	0.13	0.19 (0.14)	0.12	0.14 (0.16)	0.09
Income^b^	0.01 (0.09)	0.01	−0.02 (0.08)	−0.01	0.00 (0.08)	0.00
Scanner	−0.16 (0.11)	−0.11	−0.15 (0.11)	−0.10	−0.10 (0.12)	−0.06
*R*^2^	0.11^*^		0.14^*^		0.12^*^	

** p < 0.01

* *p < 0.0125. Regression coefficients and R2 values in bold are significant*.

a *These are factor scores from a PCA of FA values across 8 tracts*.

b *Log of total household income during wave 1 of study*.

SPSS 24 was used to calculate a semi-partial correlation between FA in the IFG-STN and d-prime as a measure of effect size (Howell, 2012). This took into account weighting but didn't take into account clustering or stratification. The semi-partial correlation was significant (*r* = 0.26, *p* < 0.001) and represents a small effect size (Cohen, 1988, 1992).

#### Controlling for the general factor

Given that FA IFG-STN was a significant predictor and FA general was a trending predictor, we tested for the specificity of the IFG-STN relationship. To do this, we ran another regression analysis predicting d-prime from FA in the IFG-STN tract while controlling for FA in the general factor tracts (FA IFG-STN and FA general were correlated at *r* = 0.67). FA in the IFG-STN remained a significant predictor of d-prime (β = 0.244, *p* = 0.035) and FA general was not a significant predictor (*p* > 0.10). The *R*^2^-values were virtually identical for the model with (*R*^2^ = 0.114) and without the general factor (*R*^2^ = 0.113), indicating that including FA general didn't significantly improve the model. See Table 4 for a summary of the regression model including both IFG-STN FA and general FA. The semi-partial correlation between FA in the IFG-STN and d-prime with FA general included in the model was significant (*r* = 0.17, *p* < 0.01), and represents a small effect size (Cohen, 1988, 1992).

**Table 4:** Regression models predicting d-prime and controlling for FA general.

	**FA IFG-STN controlling for FA general**		**FA IFG-STN controlling for FA general and psychopathology**	
	**B (SE)**	**β**	**B (SE)**	**β**
FA IFG-STN	7.40 (3.52)^*^	0.24^*^	6.61 (3.28)^*^	0.22^*^
FA General^a^	0.04 (0.10)	0.06	0.06 (0.09)	0.08
Internalizing	–	–	−0.17 (0.27)	−0.05
Externalizing	–	–	−0.13 (0.09)	−0.15
Sex	0.04 (0.11)	0.02	−0.01 (0.11)	−0.01
Age	0.04 (0.03)	0.09	0.04 (0.03)	0.09
Ethnicity	0.19 (0.15)	0.12	0.16 (0.15)	0.10
Income^b^	0.01 (0.09)	0.01	−0.03 (0.08)	−0.02
Scanner	−0.16 (0.11)	−0.11	−0.14 (0.11)	−0.10
*R^2^*	0.12^*^		0.15^*^	

* *p < 0.05. Regression coefficients and R2 values in bold are significant*.

a *These are factor scores from a PRCA of FA values across 8 tracts*.

b *Log of total household income during wave 1 of study*.

#### Psychopathology

Given that this is a community sample which was oversampled on risk for psychopathology, we wanted to confirm that presence of psychopathology wasn't driving the results. Therefore, we ran the primary analyses (d-prime predicted by FA IFG-STN, preSMA-STN, preSMA-IFG, and general) in the subset of individuals who didn't meet criteria for any diagnoses (*n* = 193). These results were consistent with those found in the full sample. FA in the IFG-STN remained a significant predictor of d-prime (β = 0.261, *p* = 0.001) and FA general was a trend level predictor (β = 0.265, *p* = 0.014). FA preSMA-STN and FA preSMA-IFG were not significant predictors (*p*s > 0.10).

In addition, psychopathology is increasingly conceptualized using dimensional as opposed to categorical approaches (Lahey et al., 2012). Therefore, we also ran analyses controlling for dimensional psychopathology by using externalizing and internalizing factor scores as covariates. FA in the IFG-STN remained a significant predictor of d-prime (β = 0.270, *p* < 0.001). FA general was also a significant predictor (β = 0.221, *p* = 0.011). FA preSMA-STN and preSMA-IFG were not significant predictors (*p*s > 0.10). The significant models are presented in Table 3. Given that both FA general and FA IFG-STN were significant predictors we tested the competing hypothesis by entering them both in the model along with internalizing and externalizing factor scores. FA IFG-STN remained a significant predictor (β = 0.218, *p* = 0.043) and FA general was not a significant predictor (β = 0.081, *p* > 0.10). This model is presented in Table 4. The *R*^2^ for this model was virtually identical with the general FA (*R*^2^ = 0.145) as without it (*R*^2^ = 0.142).

### Follow-up analyses

We ran a series of follow-up analyses in the full sample to follow-up on the primary findings.

#### Inverse efficiency

A limitation of d-prime is that it does not incorporate speed of processing. We therefore repeated the analyses using inverse efficiency as the dependent variable, which reflects the speed-accuracy trade-off between go and no-go trials. None of the FA measures were significant predictors of inverse efficiency (*p*s > 0.10).

#### Hit rate and false alarm rate

We next examined whether FA IFG-STN was related to the distinct performance variables of hit rate and false alarm rate. For hit rate, FA IFG-STN was a significant predictor (β = 0.223, *p* < 0.001). For false alarm rate, FA IFG-STN was a trend level predictor (β = −0.156, *p* = 0.077).

#### Axial and radial diffusivity

We completed follow-up analyses using AD and RD in the right IFG-STN as predictors of d-prime. RD was a significant predictor (β = −0.199, *p* < 0.01, *R*^2^ = 0.080). AD was also a significant predictor (β = 0.162, *p* < 0.05, *R*^2^ = 0.072). Given that these were both significant, we entered both into a regression as predictors of d-prime to determine if they both contributed independently (they were correlated at *r* = 0.21). Both AD (β = 0.205, *p* < 0.01) and RD (β = −0.240, *p* < 0.01) were significant predictors in this model (*R*^2^ = 0.119).

## Discussion

### Primary and competing hypothesis

We found that FA in the IFG-STN tract was a significant predictor of d-prime in the complex go/no-go task. This is consistent with previous studies on both simple go/no-go tasks and the stop signal task (SST), another task measuring response inhibition, and suggests the importance of this tract for a range of cognitive control tasks (Logan et al., 1997; Liston et al., 2006; Casey et al., 2007; Madsen et al., 2010; King et al., 2012). The present study identified a small effect size, whereas previous studies using case-control and super normal samples have generally identified an effect in the medium to large range. Overall, the current results suggest that while this DTI-performance relationship isn't driven exclusively by prior sample characteristics, the effect size may have been inflated in some cases.

In this study, general FA was not a statistically significant predictor of d-prime. Given the large sample size, this finding can be viewed as a strong null result as the power to detect a medium effect size was high (power = 0.9998). At best, general FA explained only a negligible amount of the variance in d-prime. When controlling for FA general, FA in the IFG-STN remained a significant predictor of d-prime, and adding in FA general did not improve the *R*^*2*^ of the model. This adds to the existing literature by confirming our primary hypotheses that FA in the right IFG-STN is selectively related to cognitive control in a community sample.

Given that this is a community sample with varying levels of psychopathology, we conducted several follow-up analyses that looked at the impact of psychopathology. First, we replicated our analyses in a subset of the sample that didn't meet criteria for any diagnoses. The results were the same as in the full sample. Psychopathology is increasingly being conceptualized dimensionally (Lahey et al., 2012). Thus, we also used a dimensional lens to examine the impact of psychopathology on the current results by covarying for internalizing and externalizing psychopathology factor scores. In this analysis, both IFG-STN and general FA were significant predictors of d-prime. However, when controlling for general FA and both internalizing and externalizing psychopathology, IFG-STN FA remained a significant predictor of d-prime. This provides additional confidence in the specificity of IFG-STN FA for cognitive control even when taking into account psychopathology symptoms.

It is worth noting that studies identifying a general factor of white matter have primarily been with older healthy adults or across a large age range (Penke et al., 2010; Jahanshad et al., 2013). One study in young adults with schizophrenia identified a general factor linked to processing speed (Alloza et al., 2016), although a smaller study in healthy young adults found inconsistent support for the general factor depending on the metric analyzed (Johnson et al., 2015). The present study lends support for the existence of a general factor of white matter in a young adult community sample. Its emergence in this study may in part reflect the well-powered nature of the present study relative to the smaller sample in Johnson et al. (2015). It may also reflect differences in methodology, and in particular the way in which the tracts of interest were defined. The relation of general FA to task performance in the present study was less clear, as it only reached statistical significance when controlling for dimensional psychopathology, and failed to contribute significantly to predicting d-prime when entered simultaneously with IFG-STN FA. This suggests the functional consequence of general FA to task performance is modest for this task, and potentially influenced by other variables related to psychopathology. It is important to note that the use of a large sample size as well as a range of covariates may have played a role in our ability to identify a relationship between general FA and cognitive control, especially given that this relationship emerged only when controlling for dimensional psychopathology. Continued research is needed to determine the extent to which the general factor of white matter is relevant to cognitive variables in healthy young adults as well as individuals with psychopathology.

A core challenge in the study of the brain's structural features arises in that there are a mixture of regionally-specific and more diffusely expressed features. It is thus essential to disentangle these broad vs. narrow features when examining individual differences in brain structure. The present findings highlight the utility of this combined approach to examining broad and narrow correlates of cognitive control. Further, that it is important to consider a global factor of white matter when examining cognitive processes with processing speed components, especially in samples that aren't super-normals.

### Follow-up analyses

We also completed a series of follow-up analyses in order to deconstruct our findings and provide additional specificity. Given the link between general FA and processing speed, we also examined inverse efficiency, which is a measure that captures speed-accuracy trade-offs on go and no-go trials (Townsend and Ashby, 1983). None of the FA values were significant predictors of this behavioral metric. As such, it appears that the relationship between FA in the IFG-STN and cognitive control is driven more by signal detection relative to noise rather than processing speed. However, it is important to note that inverse efficiency isn't a pure measure of processing speed, but rather is capturing processing speed in the context of inhibition.

We examined additional properties of white matter microstructure by looking at both AD (which is likely more sensitive to axonal properties) and RD (which is likely more sensitive to myelin). Both average AD and RD across the IFG-STN tract were significant predictors of d-prime, which might implicate properties of both myelin and axons. We also entered them both in as predictors, and they remained significant independent predictors. This suggests that they both contribute to d-prime. While these metrics are informative, future work should aim to incorporate more specific metrics on properties of white matter by using more advanced imaging techniques such as neurite orientation dispersion and density imaging (NODDI) (Zhang et al., 2012).

We also further deconstructed the significant d-prime relationship by examining its component processes. We looked at FA in the IFG-STN as a predictor for both hit rate (response selection) and false alarm rate (response inhibition). It was a significant predictor for hit rate and a trend level predictor for false alarm rate. This is somewhat surprising, given that previous studies have identified a relationship between response inhibition and white matter microstructure properties in this tract (King et al., 2012; Rae et al., 2015). There are several reasons why the present findings may not be consistent with those of previous studies. In this study, we used a complex version of the go/no-go task that engages multiple executive functions other than inhibition and selection (e.g., working memory). As such, false alarm rate in this task may not be as pure a measure of response inhibition as in simple versions. Also, there may be increased measurement error for false alarm rate given that there are fewer no-go than go trials. These factors may have contributed to observing a null effect for false alarm rate. However, consistent with prior studies, the present results do suggest that FA in the IFG-STN facilitates the ability to balance response inhibition and selection (Casey et al., 2007).

In contrast to prior studies, FA in the preSMA-STN and preSMA-IFG tracts weren't significant predictors of cognitive control. However, the majority of studies identifying these relationships used the SST task (Madsen et al., 2010; King et al., 2012). While the SST and GNG both measure cognitive control, the component processes are not necessarily identical: the SST probes action cancelation whereas the GNG probes action restraint (Eagle et al., 2008). Further, a meta-analysis of fMRI versions of these tasks found that while they recruit overlapping regions, they also recruit distinct regions (Swick et al., 2011). Therefore, it may be that the IFG-STN tract is relevant for both action cancelation and restraint, whereas the preSMA-STN and preSMA-IFG tracts are more relevant for action cancelation. In addition, TBSS better captures major fiber bundles, and thus is less stable in more peripheral tracts (as those studied here), and especially when they cross other fiber bundles. It may be the case that the preSMA-STN and preSMA-IFG tracts are more susceptible to crossing fibers than the IFG-STN, and thus partially explaining the null result in this present study (Jeurissen et al., 2013). That being said, a prior study using a TBSS approach identified a significant relationship in portions of these tracts (King et al., 2012).

## Limitations

While this study has a number of strengths including a large diverse sample, it is important to note a few limitations. One potential limitation is that we used a complex version of the go/no-go task. While this version is often employed because of its high executive function component, it may index multiple processes (e.g., working memory) that are less specific to response inhibition. This seems particularly likely given that observed relations were stronger for d-prime and hit rate compared to false alarm rate. Another limitation is the use of a twin sample, which required reducing our statistical power to account for clustering. However, even when accounting for the presence of twin pairs, the sample size was large, which is in contrast to previous neuroimaging studies which have traditionally been underpowered.

There are also a few limitations to note in regards to the analytic approach to the neuroimaging data. While we corrected for eddy currents, we didn't correct for EPI distortions, which could impact frontal tracts. Future studies should attempt to circumvent this by using sequences more robust to these distortions such as acquiring both a bottom-up and top-down image in k-space for each diffusion gradient (Andersson et al., 2003). However, that we found effects in the IFG-STN tract likely indicates that the EPI distortions didn't substantially influence the results. Further we utilized TBSS because several other studies on white matter and cognitive control used this technique (thereby allowing for more direct comparisons), and because it allows for examination of the center of tracts and thus reduces the problem of partial volume effects (Smith et al., 2006; Madsen et al., 2010; King et al., 2012). However, there are some limitations to this technique such as that it sometimes fails to identify the center point of the tract and may introduce additional confounds by aligning subjects to a common template before extracting microstructure values rather than extracting them in native space. Slight inconsistencies between tractography masks and skeletonized masks may also produce discrepancies. These discrepancies arise in part because of different alignment techniques. Future studies should confirm these findings by extracting values in native space and comparing them with the present results. In addition, it should be noted that we focused on properties of microstructure, but macrostructure properties may also play a role here and should be further investigated. Finally, there are known limitations to DTI imaging, such as difficulties modeling crossing fibers, and future studies should use more advanced techniques (e.g., Q-ball imaging) that better take into account these limitations to determine if the results still hold (Tuch et al., 2003; Jeurissen et al., 2013; Jones et al., 2013).

## Ethics statement

This study was carried out in accordance with the recommendations Vanderbilt University's Institutional Review board and all research was in line with its human subjects' policies. All subjects gave written informed consent in accordance with the Declaration of Helsinki.

## Author contributions

NDW, BAL, BBL, VV-G, KEH, and DHZ designed research; KEH, VV-G, and KBW performed research; KEH, BBL, BA, BDB, VV-G, BCY, AJP, and BAL analyzed data; KEH and DHZ wrote the paper.

### Conflict of interest statement

The authors declare that the research was conducted in the absence of any commercial or financial relationships that could be construed as a potential conflict of interest. The reviewer NPB and handling Editor declared their shared affiliation.
